# Heart Rate Estimation from Incomplete Electrocardiography Signals

**DOI:** 10.3390/s23020597

**Published:** 2023-01-04

**Authors:** Yawei Song, Jia Chen, Rongxin Zhang

**Affiliations:** 1 School of Electronic Science and Engineering (National Model Microelectronics College), Xiamen University, Xiamen 361005, China; 2 Innovation Laboratory for Sciences and Technologies of Energy Materials of Fujian Province (IKKEM), Xiamen 361005, China; 3 Key Laboratory of Underwater Acoustic Communication and Marine Information Technology, Xiamen University, Ministry of Education, Xiamen 361005, China

**Keywords:** heart rate, electrocardiography, neural network, short time signal, informative missingness

## Abstract

As one of the most remarkable indicators of physiological health, heart rate (HR) has become an unfailing investigation for researchers. Unlike many existing methods, this article proposes an approach to implement short-time HR estimation from electrocardiography in time series missing patterns. Benefiting from the rapid development of deep learning, we adopted a bidirectional long short-term memory model (Bi-LSTM) and temporal convolution network (TCN) to recover complete heartbeat signals from those with durations are less than one cardiac cycle, and the estimated HR from recovered segment combining the input and the predicted output. We also compared the performance of Bi-LSTM and TCN in PhysioNet dataset. Validating the method over a resting heart rate range of 60–120 bpm in the database without significant arrhythmias and a corresponding range of 30–150 bpm in the database with arrhythmias, we found that networks provide an estimated approach for incomplete signals in a fixed format. These results are consistent with real heartbeats in the normal heartbeat dataset (γ > 0.7, RMSE < 10) and in the arrhythmia database (γ > 0.6, RMSE < 30), verifying that HR could be estimated by models in advance. We also discussed the short-time limits for the predictive model. It could be used for physiological purposes such as mobile sensing in time-constrained scenarios, and providing useful insights for better time series analyses in missing data patterns.

## 1. Introduction

Heart rate (HR) is an important physiological indication of human beings that reflects the variety of psychological conditions to a considerable extent. Specifically, HR is a direct metric of how healthy the cardiovascular system is, hence, measuring and estimating HR is crucial in many aspects, including public health oversight, sleep monitoring, and control of exercise intensity. Because HR fluctuates substantially with psychological aspects, it is widely used in lie detection, sleep monitoring, fatigue driving, and so on [1,2,3]. In general, we estimate HR by electrocardiography (ECG) in the clinic, which is measured by sensors that capture weak electrical activity generated by the cardiac cycle when the heart beats. ECG signals have been of great interest to researchers [4,5,6], because they include noticeable temporal features for human physical and psychological analysis under different circumstances. The explorations for heartbeats and ECG morphological features provide vital biomedical information for human status. However, deep knowledge and long-term records are necessary for signal interpretation. Simultaneously, it is nearly impossible to avoid committing personal errors in the analytical process in real situations such as empirical mistakes. As a result, computer-assisted methods for ECG analysis have piqued the interest of researchers [6,7,8].

Researchers have made significant advances in computer vision and natural language processing as a result of the success of deep learning (DL). Convolutional neural networks (CNN) and recurrent neural networks (RNN) have recently pulled significantly ahead of a variety of research scopes with the advantage of processing images and text [9,10]. Simultaneously, an increasing number of researchers are focusing on HR signal estimation using DL methods. Various approaches to measuring HR using artificial neural networks (ANN) have been proposed [11], and the majority of the methods contribute to reducing the noise in order to obtain an accurate HR number. A considerable number of researchers focus on the automatic pathological classifications of ECGs [12,13], and there are still a few researchers working on improving the performance of conventional methods. Many existing methods for pulse estimation with DL used time-frequency representation, achieving good performance in public or private databases. However, the frequency domain approach relies on the accurate representation of long-time domain signals. In general, the classic time window for estimating the instantaneous heart rate (iHR) with frequency domain analysis is 3–4 cycles. Meanwhile, we have to wait for the appearance of two adjacent R-peaks in the most distinguishing ECG if we want to get iHR using the time domain method, which is also known as the R–R interval calculation (RRI). Thus, theoretically, acquiring the iHR necessitates at least one cardiac cycle.

The global community has suffered a massive impact resulting from the coronavirus disease 2019 (COVID-19). The detection of physiological indices has recently received a lot of attention from academics [14,15]. Because of the toxicity and the transmissibility of COVID-19, people are increasingly concerned with their physical condition. Physiological indicator monitoring is a reliable and simple method for health surveillance. However, most of the realizations take too long to assess the temperature or other indicators [15]. For example, the remote photoplethysmography (rPPG) method for heart rate detection is based on a long-term stationary facial video [16,17], wherein interferences resulting from the movement or the varying illumination are unavoidable and nonnegligible. This kind of time-consuming method is not applicable in many public and crowded places; therefore, short-term physiological index (e.g., temperature, heart rate, and respiratory rate) measurements are especially important in this case, which would make an invaluable contribution to public health security.

In this paper, we propose a method for fast-estimating iHR during a very short time window, and it does not require HR signals that last throughout the whole cycle in the time dimension. No one has ever discussed these things, and it is the first work to estimate iHR in an extremely short time series, with even less than one cycle in the time domain. Although we can obtain a long period of ECG recording easily, the research for short time HR estimation still has some implications. On the one hand, less time means faster corresponding signal processing and less data transfer or storage. This may have great significance for real-time monitoring systems. On the other hand, HR measurement depends on contact and stable attachment of sensors usually. It is inevitable that the quality of signals will be damaged during measurement or transmission, and HR measurement in missing data patterns would be beneficial for physiological perception in this case. We endeavor to develop an instantaneous and adaptive pulse estimation system using DL. CNN and RNN are used to address the issue of informative missingness [18].

We investigate the shortest data length that HR can recover from fragmented heartbeat signals, and evaluation metrics for results analysis are also presented. Experimental results on ECG datasets show that our method estimated HR in a very short time, and we seek the limit of time. Future projects and directions are also discussed.

The rest of the paper is organized as follows: In Section 2, related HR estimation works are described; in Section 3, the detail of the proposed approach is presented; then, the experiment setting and results in heartbeat database with normal sinus rhythm and database with arrhythmias are discussed in Section 4. Finally, we conclude the paper in Section 5.

## 2. Related Works

There are already some academics using ML or DL to carry out practical applications such as biology, meteorology, etc. For instance, Hannun et al. [13] proposed a deep classification model that helps clinicians diagnose heart disease. Song et al. [19] proposed a novel transfer learning scheme using CNNs to pretrain the HR estimator. They constructed a featured map with noise-contaminated pulse signals extracted by conventional rPPG methods. Qiu et al., proposed a framework that integrated Eulerian video magnification (EVM) [17,20] and DL to achieve iHR estimation. In their experiment, the performance of the short-time HR estimation was evaluated in different time windows. As the window size grows, the performance improves steadily in all aspects, and the approach maintains good performance in real-time HR estimation with the shortest window size, i.e., 4 s.

Many researchers have discussed DL techniques in time-series prediction [21,22,23], which are critical in many practical applications including health care, meteorology, and traffic flow forecasting. Their works indicate that a deep model not only captures the long-term temporal dependencies in the time series, but also allows the missing patterns to achieve better prediction results. More specifically, Alharbi A et al. [24] used RNN, long short-term memory (LSTM), gated recurrent units (GRU), and bidirectional long short-term memory (BI-LSTM) using one layer, two layers, and three hidden layers for heart rate forecasting. Comparing those DL models on different scenarios: a lag of 3 min and forecasting 5 min in advance, a lag of 5 min and forecasting 7 min in advance, and a lag of 10 min and forecasting 15 min in advance, respectively. The model that has the lowest RMSE would be chosen to predict HR. Biswas et al. [25] combined CNN and LSTM to predict HR from PPG signals contaminated by various motion noises. Dunn et al. [26] used vital signs collected by wearable sensors to predict several clinical measurements. The results demonstrate that the accuracy of HR prediction hinges on the continuous and longitudinal assessment of physiological monitoring [27].

It is known that the length of time and ratio of missing data plays a vital role in the performance of the DL models. However, it is hard to ensure the time continuity of rPPG or HR signals. Furthermore, only a few studies on short-time HR estimation have focused on the shortest duration of the input hear-rate signal of 1s (at least 1 cycle); therefore, we try to propose a method for HR estimation on smaller time scales.

## 3. Methods

**Figure 1:**
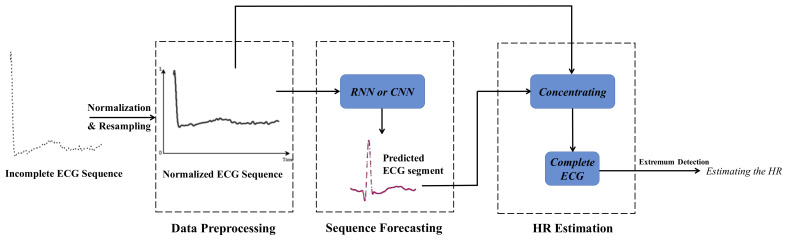
The diagram of the proposed iHR estimation approach employing time-series forecasting with CNN and RNN, and its main three steps: data preprocessing, sequence forecasting, and HR estimation. Following ECG signal normalization, a pretrained neural network would be able to generate time series for a short period of time in the future. We calculate a complete RRI and estimate HR in advance by combining the restored and original signals.

The proposed iHR estimation approach is based on CNN and RNN. The whole architecture of the model is presented in Figure 1. The proposed iHR estimation method adopts time series forecasting with CNN and RNN. In the data preprocessing module, ECG signals are normalized to a uniform amplitude range and then resampled. CNN and RNN are applied to forecast the missing ECG segment in the next module. We can estimate HR in a very short time period by concentrating on the normalized input ECG segment and the predicted output ECG segment.

Conventional RNNs have had great success in sequence tasks. However, recent researches indicate that convolutional sequence modeling outperforms recurrent architectures in a wide range of tasks and datasets. In this paper, we focus on measuring problems based on an incomplete time series with a fixed pattern, i.e., the realization of the short-time HR estimation. In order to achieve this goal, the bidirectional long short-term memory model (Bi-LSTM) and temporal convolution network (TCN) has been evaluated, respectively. Since the input sequence is too short to reveal the periodicity of HR signals, it is inapplicable to process the input sequence with traditional Fourier transform (FT) or wavelet transform (WT). In addition, it is hard to omit the missing data when the missing rate is high and we want to ensure adequate performance. Using the parameter-sharing mechanism of RNN, time-series prediction and other related works have been discussed in [28,29]. RNNs for missing data have been studied in [23] and applied to health care and biology. Recently, a convolution network with simple architecture was proposed for sequence modeling in [30]. In order to address the missing data problem in time series analysis, we filled in the missing values with substituted values, which are estimated by neural networks. It is well known that the performance of DL models is correlated with different input lengths and the portion of the missing data, thus, the effects of these factors will be evaluated. Data preprocessing, which is utilized to improve the model’s performance, is also discussed in this paper.

### 3.1. Bidirectional Long Short-Term Memory Model (Bi-LSTM)

Deep neural networks (DNNs) would be able to approximate arbitrary function mappings if there are unlimited data and computing power. Given an input matrix X, each column (denoted as x) is fed to trained models one by one to yield a specific output (denoted as y). However, the procedure ignores the context connection of the input (i.e., the relationship between the columns of X). RNNs were introduced to exploit the correspondence of the input sequence in the time dimension. To achieve memorization and learning from location information, RNN neuron outputs are recursively connected to their own neuron inputs through the self-connection of hidden layers, capturing sequential information.

Although RNNs have been successfully applied to time series data analysis and machine translation, the structure cannot be applied to learn long-term dependencies because of the problem of gradient vanishing [31], leading to deficiencies of RNNs in long-distance time series analysis. LSTM was proposed to mitigate the problems of gradient vanishing and explosion by adding mechanisms of channel gating into traditional RNN units [32]. In addition, Bi-LSTM further improves the adaptability to changes in the time dimension by combining the forward LSTM and the backward LSTM.

**Figure 2:**
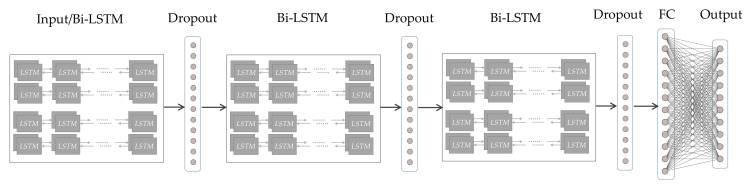
System structure of the iHR estimation model using Bi-LSTM. The original signal as input for Bi-LSTM, and hidden units of LSTM capture temporal dependencies in time series. Dropout layer close to each Bi-LSTM layer to reduce overfitting. To generate HR estimation sequences, three layers of Bi-LSTM and dropout, an FC layer, and an output layer were stacked.

Figure 2 presents the adopted Bi-LSTM model with its hierarchical structure (from left to right). Introducing the dropout behind the Bi-LSTM layer contributes to preventing overfitting in training [33], which is one of the most serious problems in deep models. Randomly dropping out could avoid excessive concentration of weights on certain neurons and enhance the stand-alone capability of each unit to some extent. As shown in Figure 2, by stacking three layers of Bi-LSTM and dropout alternatively, then flattening the output of multiple layers and using a fully connected layer (FC) to generate time series data in the end, we can predict the signal trend in a short time. With two adjacent R peaks of the fragmentary signal recovered, the iHR could be measured by peak detection algorithms.

### 3.2. Temporal Convolution Network

Conventional wisdom thought that CNNs will be appropriate for image processing, during which the image features would be extracted by convolution kernels gradually. Recently, Lea et al., presented long-range temporal patterns that could be captured by CNNs by using a hierarchy of temporal convolutions networks (TCN) [34]. Bai et al., pointed out that TCN is equivalent to a 1D fully-convolutional network combined with causal convolutions [30]. A residual connection was repeatedly applied in TCN for learning modifications and stabling gradients so that degradation problems in very deep networks have been alleviated [35]. A residual block contains a branch leading out to a series of transformations F, whose outputs z are added to the input x of the block:

(1) z=Activation(x+F(x))

**Figure 3:**
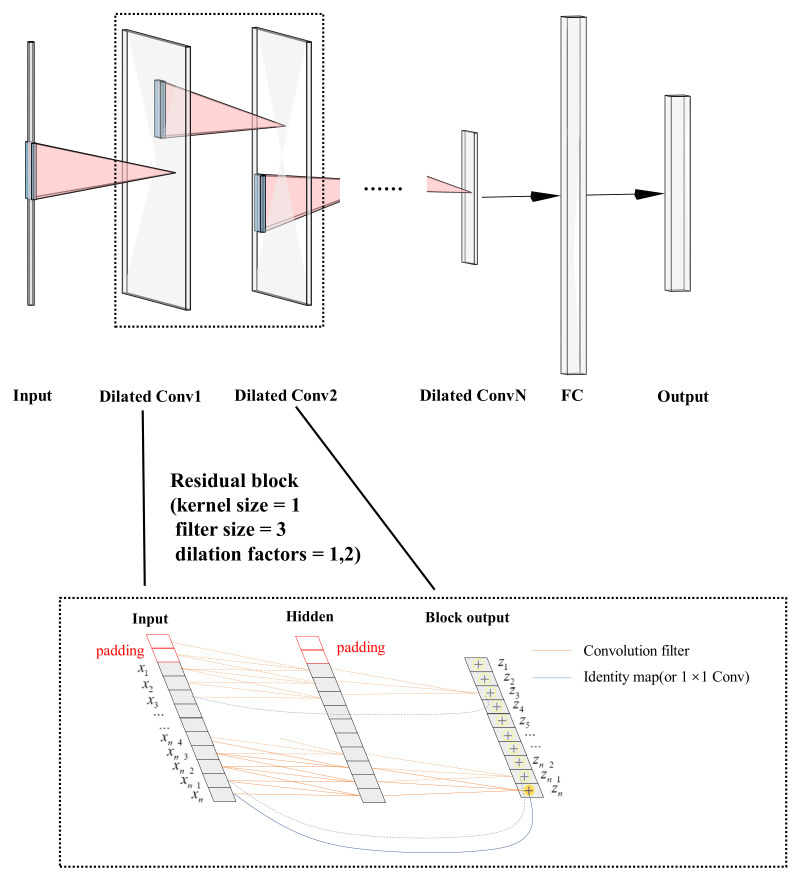
The system structure of the iHR estimation model using TCN in AlexNet style. Stacking residual blocks with dilation casual convolution layers using skip connection (simplified and visualized by the black dash boxed areas), a generic TCN model would be designed for sequence modeling. The bottom boxed areas show an example of a residual connection in a TCN. Two solid black lines indicate that the input of the Dilated Conv1 module in the top boxed area corresponds to the input layer in the bottom boxed area, and the input of Dilated Conv2 module in the top boxed area corresponds to the block output layer in the bottom boxed area. In this example, a dilated causal convolution with dilation factors d = 1,2 and filter size k = 3, the origin lines are filters in the residual function, and the blue lines are identity mappings. Across layers via identity mappings, skip connections effectively mitigate the gradient problem in deep models. To generate forecasting sequences, we also use an FC layer and output layer.

The basic structure of the TCN model with residual blocks is shown in Figure 3. Simplified residual blocks have two dilated causal convolution modules and a shortcut for skip connection, which are cascaded. Similarly, the dropout layer is next to the Conv1D layer to overcome the overfitting in deep model training. With the network depth increasing, the number of filters drops gradually while the dilation rate grows exponentially in the residual block. Standard TCN architecture requires that the length of input signals is equivalent to the length of output signals. The most common way to change the length of the input sequence is zero padding, and we used fully connected layers to receive outputs of the last residual block so that the network could accept variable lengths of input sequences. The model is flexible for the ability to process the sequence at different timescales.

### 3.3. Data Preparation for Short-Time Heart Rate Estimation

Unlike traditional iHR measuring approaches, our input time series data were extremely short and less than one cardiac cycle, meaning that features in the frequency domain did not exist at all. In this way, time series analyses provide useful insights for iHR estimation. The fastest estimation of HR in real life is to calculate the R–R interval in the time domain (the time period between the successive R peaks) because the magnitudes of the R waves are much greater than others. It seems that the input signal can be divided into peaks and non-peaks simply, and only the R peaks need to be covered for HR measuring. However, it might not work as it ignores the hidden nature of the information in ECG. In view of biometric features such as the length of P waves, the shape of P waves and the time period between S wave and R wave, it is necessary to take into consideration the original waveform of the signal.

Data preparation has a huge impact on data analysis. In this paper, the input and output of network models were designed with 1D signals because 1D model speeds up the training process for little computing burden. The 1D ECG signal is normalized between unified lower and upper bound, and resampled within a range $\left[f_{s1},\;f_{s2}\right]$. The resampled signal is divided into several equal-length segments using a fixed window. Each segment will not overlap in the time domain without leveraging any heartbeat location information. Meanwhile, the long duration of exception records will be removed to improve the model’s performance [36].

**Figure 4:**
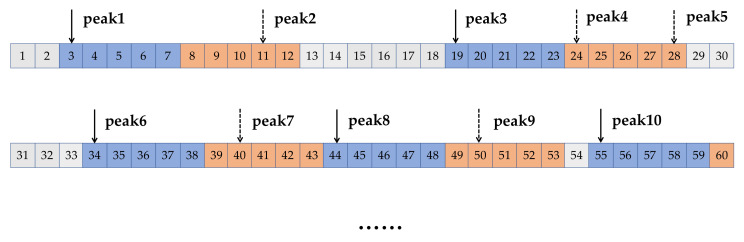
Examples of data segmenting of the recorded ECG. The time-leading parts are displayed in blue and the time-lagged part are displayed in orange. The elements between the time-leading parts and the time-lagged parts (grey) are deleted.

Each segment was divided into two clips additionally, naming the time-leading part and time-lagged part, as shown in Figure 4. In fact, the lengths of the two clips could be unequal. Setting the length of the time-leading part to a small value ensured that there was no more than one complete heartbeat and the time-leading part in the segments were directly used as the inputs of the model. Without intricate data preprocessing, we hoped that the models would make full use of the characteristics of time series in the time domain.

In the next data processing step, the dropout mechanism was introduced in different layers. Specifically, we used DL models trained by the time-leading parts to predict the time-lagged part. Each time-lagged part, as the neural network’s output, was also less than a cycle. A complete cardiac cycle could be recovered by combining the output with the input of the network. The proportion of the input sequence taking in the concentrating time series is denoted *α*, then the proportion of the predicted output sequence taking in the concentrating time series is 1 − *α*, where α∈[0,1]. Obviously, with *α* increasing, the performance of these models in iHR estimation increased progressively. In other words, a higher missing rate leads to a lower accuracy for data complement.

**Figure 5:**
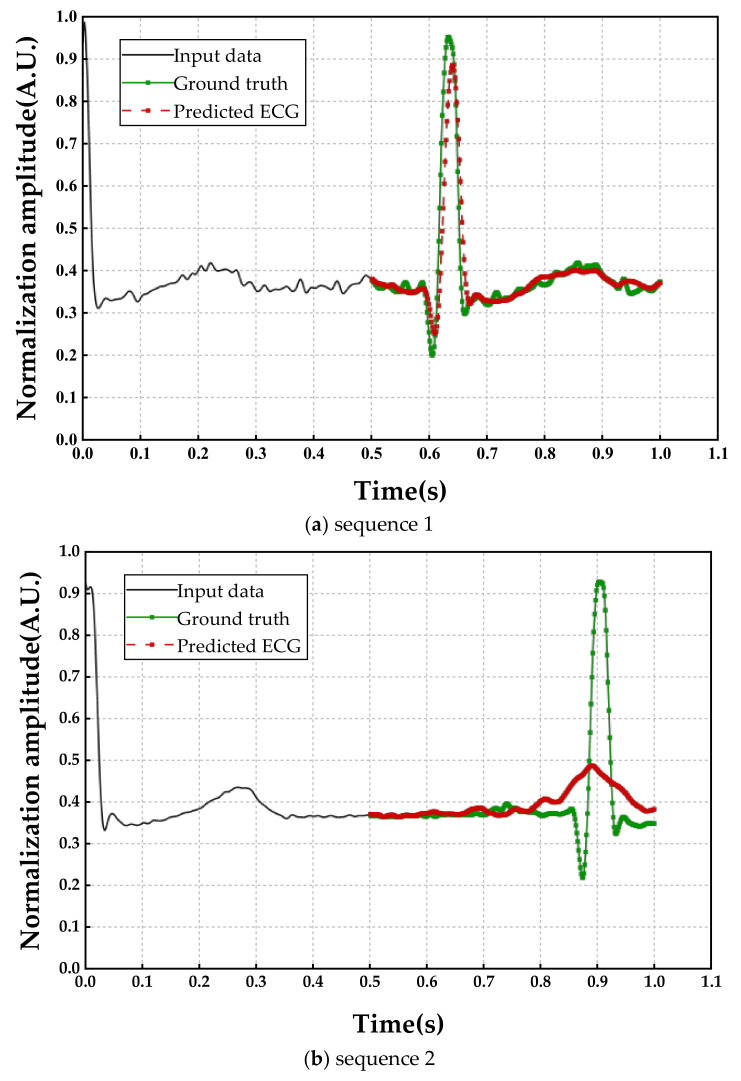
Prediction results of two sequences (**a**,**b**). Black lines denote the input sequence and green dash lines denote the ground truth ECG. The red imaginary lines denote the predicted ECG from the proposed method. The change in shape does not affect the determination of the extremum point.

It is noteworthy that there are always errors between the real ECG and the predicted ECG, which is inevitable. The magnitudes of R peaks in ECG are much larger than the others, thus, they are easily regarded as noises in DL networks. As shown in Figure 5, deformations have occurred in the predicted R peaks. The degree of deformation is related to the proportion of the missing data in the whole cardiac cycle. However, we do not know the ratio of the missing data because the full cycle duration is exactly unknown. Fortunately, HR values could be measured by the extremum detection algorithm.

We estimated iHR from the recovered segment combining the input and the predicted output, with the calculation expressed as follows:

(2) $$\mathrm{HR}=\frac{1}{T_{\mathrm{RRI}}}\;\times \;60$$

where T_RRI_ denotes the time duration of RRI, and HR was measured in beats per minute (bpm).

## 4. Evaluation Metrics and Result Analysis

The proposed iHR estimation model has been evaluated on the MIT-BIH Normal Sinus Rhythm database, which belongs to the PhysioNet database [37]. This is a publicly available collection of ECG 1D data from 5 men, aged 26 to 45, and 13 women, aged from 20 to 50. Eighteen long-term ECG recordings are included in the database without significant arrhythmias. As described earlier in Figure 4, we obtained more than 50,000 segments for each subject after window segmentation processing, where each segment is further divided into the time-leading part and the time-lagged part.

For patients with heart disease (ventricular tachycardias, heart failure, ectopic runs, etc.), ECG may be contaminated if there are position changes or interference signal superposition through the measurement process. Meanwhile, arrhythmias may lead to a wider HR range for those patients. Therefore, in order to evaluate our approach more thoroughly, a database, named “A Large Scale 12-lead Electrocardiogram Database for Arrhythmia Study” [38,39] was also chosen for performance examinations. This database also belongs to PhysioNet [37], containing ECGs from 45,152 patients under resting conditions and 64 different types of arrhythmias. The sampling rate is 500 Hz, and each record in the database lasts for 10 s. After the aforementioned window segmentation processing, we obtained more than 360,000 segments from the whole database.

The proposed iHR estimation approach has been implemented using TCN and Bi-LSTM, which are developed using Tensorflow and Keras. In terms of the training setup, these sequences are divided into training and testing subsets with a partition ratio of 3:1. We also employed the Adam optimizer with a batch size of 64, a learning rate of 0.001, and a small learning decay. The maximum epoch number was 1000, and the training would stop if the validation loss did not decrease in 5 epochs. The algorithm was implemented using Python 3.7, with CUDA enabled NVIDA Tesla P100 GPU and NVIDA Geforce RTX3060 GPU.

Of course, the measured iHR is frequently not numerically equal to the real one, and it is not appropriate to define model performance by accuracy. As a result, additional metrics for performance evaluation were introduced. For performance evaluation, we used the following metric based on HR estimation errors, which is the difference between the predicted HR and the real HR:

(3) $$M_{e}=\frac{1}{N}\sum_{i=1}^{N}{\mathrm{HR}}_{e}(i)$$

where N denotes that the number of cardiac cycle numbers, and

(4) $${\mathrm{HR}}_{e}(i)={\mathrm{HR}}_{p}(i)-{\mathrm{HR}}_{\mathrm{gt}}(i)$$

where the subscript e, p, and t denote the error, the prediction, and the ground truth, respectively, and i indicates that the variable is related to the i-th segment.

The dispersion of HR_e_ was evaluated by its standard deviation (denoted as HR_SD_):

(5) $${\mathrm{HR}}_{\mathrm{SD}}=\sqrt{\frac{1}{N}\sum_{i=1}^{N}{\left({\mathrm{HR}}_{e}(i)-M_{e}\right)}^{2}}$$

A low value of HR_SD_ indicates that the measuring values of HR_e_ tend to be closer to the average value.

We used the mean absolute percentage error (denoted as MAPE) to evaluate the accuracy of the sequence model with the following expression.

(6) $$\mathrm{MAPE}=\frac{1}{N}\sum_{i=1}^{N}\frac{\left|{\mathrm{HR}}_{e}(i)\right|}{{\mathrm{HR}}_{\mathrm{gt}}(i)}\;\times \;100\%$$

where a lower value indicates that the predicted values are closer to the ground-truth ones.

The differences between the values predicted by an estimator and the observed values were evaluated by the root mean square error (denoted as RMSE), which is calculated as follows.

(7) $$\mathrm{RMSE}=\sqrt{\frac{1}{N}\sum_{i=1}^{N}{\left({\mathrm{HR}}_{p}(i)-{\mathrm{HR}}_{\mathrm{gt}}(i)\right)}^{2}}$$

where RMSE∈[0, ∞), and a lower value indicates that there are fewer outliers. RMSE is strongly affected by outliers.

We used the Pearson correlation coefficient (denoted as γ) to measure the linear correlation between the predicted HR and the ground truth value.

(8) $$\gamma =\frac{\sum_{i=1}^{N}\left({\mathrm{HR}}_{p}(i)-M_{p}(i)\right)\left({\mathrm{HR}}_{\mathrm{gt}}(i)-M_{\mathrm{gt}}\right)}{\sqrt{\sum_{i=1}^{N}{\left({\mathrm{HR}}_{p}(i)-M_{p}\right)}^{2}}\sqrt{\sum_{i=1}^{N}{\left({\mathrm{HR}}_{\mathrm{gt}}(i)-M_{\mathrm{gt}}\right)}^{2}}}$$

where M_p_ denotes the mean value of predicted HR, and M_gt_ denotes the mean value of the ground truth values. γ∈[−1,1], where 1 indicates the total positive linear correlation and -1 indicates the total negative correlation.

### 4.1. The Result with Different Input Lengths

The accuracy was conditioned by the sampling frequency of signals because it determines the resolution of HR and influences the model’s abilities. Though higher sampling frequency means a higher accuracy rate, longer-term dependencies needed to be captured. Furthermore, longer sequence modeling often requires more parameters, resulting in more iteration epochs and longer convergence time. The original sampling frequency of ECG (denoted as $f_{s}$) was 128 Hz, and we resampled the signal at various ranges of frequencies, i.e., 64 Hz, 128 Hz, 256 Hz, 384 Hz, 512 Hz, 640 Hz, 768 Hz, 896 Hz, and 1024 Hz, corresponding to 0.5$0.5f_{s},f_{s},2f_{s},3f_{s},4f_{s},5f_{s},6f_{s},7f_{s},\mathrm{and}8f_{s}$ respectively. We uniformly sampled on recorded ECG in the time domain during downsampling. Upsampling was accomplished using the cubic spline interpolation function, and cubic polynomials were used to generate smooth curves. Profiting from the respective network architecture of CNN and RNN, we deepened the TCN while widening the Bi-LSTM with the growing length of the input sequences.

As shown in Table 1, we experimentally demonstrated the performance of CNN and RNN with *α* = 0.5 (using the lag of 0.5s to predict ECG in the next 0.5 s in advance). We evaluated the test set results using five metrics. To better evaluate the influence of the sequence length and to find the best sampling time, we kept the limits of signals within the range from 60 bpm to 120 bpm, which is within the normal sinus heart rate range. Because the first R peak is fixed at the input signal and the next R peak is fixed at the output signal, the incompleteness of the input signal and the integrity of the predicted signal are guaranteed.

Compared with Bi-LSTM, TCN has a deeper layer framework which connects input, hidden layer, and output. Correspondingly, it is possible to have a better performance in model fitting, and it does provide better predictive properties in all indicators. In addition, simpler 1D convolutional architecture was used to capture characteristics of the time series by causal convolution, reducing the computation burden greatly; however, the forecasting performance gap between them in the experiment was not obvious. RNN has many characteristics, such as clear status, better generalization capabilities, and so on, and it is, thus, more suitable for transfer learning. As a result, evaluating CNN and RNN is equally important and should not be mutually exclusive.

Although the sampling frequency varies over a large range (64–1024 Hz), M_e_ of TCN and Bi-LSTM fell in a small range and do not show a significant regularity (see Table 1). However, three metrics (i.e., HR_MAPE_ and HR_RMSE_) show a general trend of decreasing and then increasing, corresponding to a trend of increasing and then decreasing in performance, which indicates that the variations of the sequence length is distributed over an interzone with a higher accuracy. It should be noted that all γ were greater than 0.9, indicating that estimated HR has a strong linear correlation with real HR. TCN method performs best at the sampling frequency of 640 Hz and Bi-LSTM method performs best at the sampling frequency of 128 Hz. Apparently, both models show outstanding performance in time series with missing values, and the prediction result is not determined by the length of sequence.

### 4.2. The Result at Different Input Duration Time

Although HR can be estimated from the predicted signals, the impact of the deformed shape of the predicted signals cannot be neglected. In fact, R peaks would be well restored if the input sequence accounts for a large proportion (usually when *α* > 0.7). As shown in Figure 5, where the horizontal axis represents the time (with 1/640 s as the time interval) and the vertical axis represents the normalized ECG amplitude. The input sequence 1 in Figure 5a and an equal length of the input sequence 2 in Figure 5b were drawing as black lines, and they were similar in magnitude and shape, but significant differences in the output signals (red lines), as seen. As input for two signals that were waiting to be made up, we observed that the waveform had been restored perfectly compared with the ground truth in Figure 5a, while the magnitude of the R peak was obviously lower than the real signal in Figure 5b. The reason for this phenomenon is that the first input sequence accounts for a higher proportion of the cardiac cycle (i.e., a higher α), and the output performance depends heavily on the relative length of the input. In other words, the higher percentage of input sequence shared, the better results of the model prediction. However, we really do not know exactly what percentage of this mutilated signal, and it is widely believed that HR is easily changed from a physical and psychological condition.

**Figure 6:**
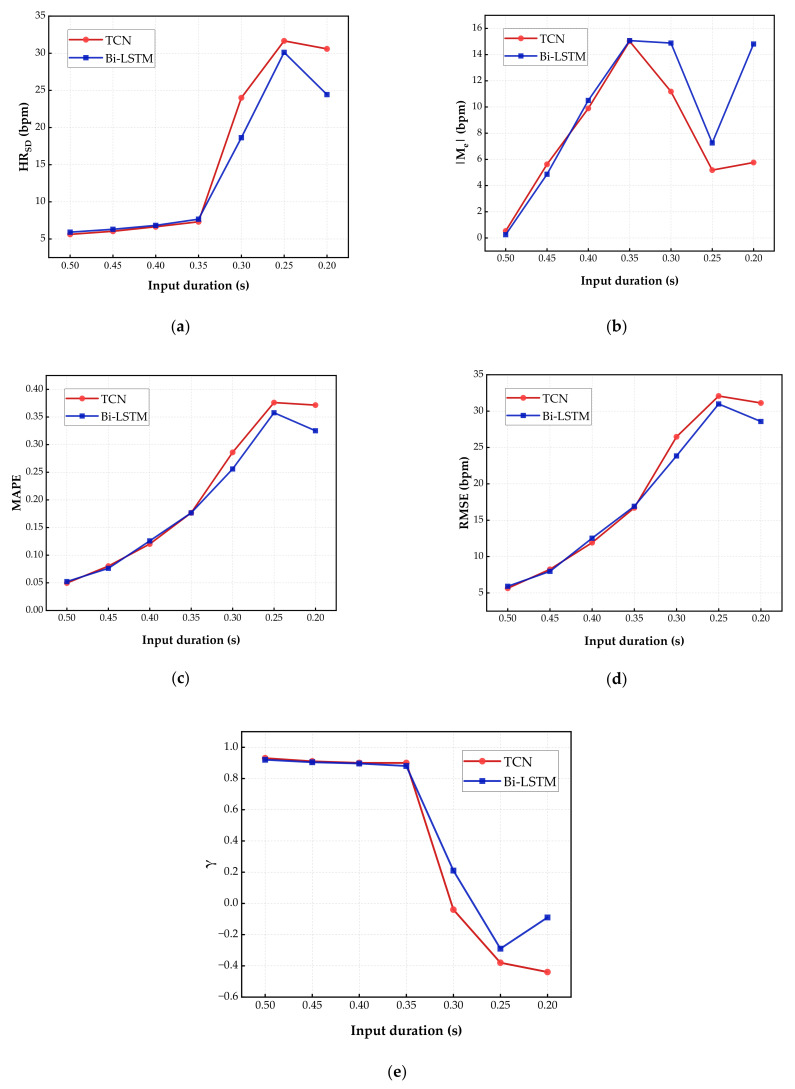
Results comparison on inputs of different lengths. (**a**) The absolute value of mean error; (**b**) standard deviation; (**c**) mean absolute percentage error; (**d**) root mean square error; and (**e**) Pearson correlation coefficient. The red line denotes the predicted results of CNN models and the blue line denotes the results of RNN models. As the time duration of input fell from 0.5 s to 0.2 s, a high, similar trend was demonstrated in Bi-LSTM and TCN.

It is necessary to discuss the effects caused by variations of the input length. A higher input length (i.e., a higher *α*) results in a better performance, however, leads to higher computing complexity. Therefore, we need to explore a trade-off between the performance and the input length for the proposed approach. The result of this experiment is presented in Figure 6, where CNN and RNN are compared at a sampling rate of 640 Hz and 128 Hz, respectively, which have the lowest HR_SD_, HR_MAPE_, and HR_RMSE_ in previous experiment. RNN was stacked by LSTM layers (see Table 2) and CNN was stacked by residual blocks (see Table 3). Meanwhile, we have described further details about the networks, including the number of neurons, dropout rate, and so on.

It should be noted that, to the best of our knowledge, our work is the first attempt to estimate HR in less than a cycle, and few studies have explored the possibility of using a short-time input sequence to predict a longer output sequence. Obviously, higher accuracy when inputting longer time series, task outcomes are always limited by the inputs. We are interested in the extreme predictive capacity of DL models, i.e., predicting heart rhythm based on the shortest and incomplete ECG signals. Comparing results (as in Figure 6) for inputting 0.5 s ECG segment and forecasting the following 0.5 s, inputting 0.45 s ECG segment and forecasting following 0.55 s, inputting 0.4 s ECG segment and forecasting following 0.6 s, inputting 0.35 s ECG segment and forecasting following 0.65 s, inputting 0.3 s ECG segment and forecasting following 0.7 s, and inputting 0.25 s ECG segment and forecasting following 0.75 s.

From the results with different input lengths, as the time duration of input decreases, the absolute value of M_e_ climbed up initially, followed by a decline, and remained steady; lastly, 0.35 s and 0.25 s were two critical turning points in the curves. Meanwhile, HR_SD_, HR_MAPE_, and HR_RMSE_ rose slowly with the decreasing percentage of input in the early stage. Three metrics rose rapidly after the input duration was reduced to 0.35 s. Continuing the reduction of the input duration to 0.25 s, HR_SD_, HR_MAPE_, and HR_RMSE_ began to remain stable. In terms of the Pearson correlation coefficient γ, all results were greater than 0.8 until the input duration was below 0.35 s, proving a strong positive linear correlation between input and predicted output. Within the range of 0.3–0.25 s, the absolute value of γ is lower than 0.3, meaning that there was a weak linear correlation between input and estimated HR. The Pearson correlation coefficient also maintains steady in the later intervals. Therefore, the fastest HR estimation should be maintained input sequences of no less than 0.35 s duration. In order to implement normal resting HR quick estimation at an acceptable accuracy, the duration of input sequences should be no less than 0.35 s.

### 4.3. The Result in Arrhythmia Cases

In this section, A Large Scale 12-lead Electrocardiogram Database for Arrhythmia Study was used to evaluate the performance of the proposed approach. It should be noted that signals from the MIT-BIH Normal Sinus Rhythm database were clean and steady. Without severe noises, a normalized signal with a small measurement error can be inputted directly into DL models. Compared with the MIT-BIH Normal Sinus Rhythm database, signals from A Large Scale 12-lead Electrocardiogram Database for Arrhythmia Study were polluted by serious interference. Therefore, ECG noise reduction was needed to be applied and the former option of selecting a heart rate of “60–120 bpm” was naturally less effective.

For 12-lead ECG, each channel of the ECG varied considerably in amplitude and base-line. We merged all the channels to one by taking their average. Then, a three order Butterworth low pass filter was used to remove the signal noise with the cut-off frequency of 75 Hz. Although high-frequency noise was suppressed, base-line drifting and motion or muscle interferences continued to exist in the ECGs. Subsequently, we adopted a fast non local means (NLM) algorithm to further handle the signal. Accordingly, the denoised signal S(i) was expressed as [40].

(9) $$S(i)=\frac{1}{Z(i)}\;\sum_{t\in N(i)}\omega (i,t)v(t)$$

where N(i) is a certain “search neighborhood” of *i*, v(t) is the original ECGs, $Z(i)=\sum_{t}\omega (i,t)$, and *ω* is the weighted coefficient with the calculation given as follows:

(10) $$\omega (i,t)=\exp(\frac{\sum_{\delta \in \Delta }\left(v(i+\delta )-v(t+\delta )\right)}{\sqrt{2n}\lambda })$$

where Δ represents a local patch of surrounding samples, *n* is the number of samples, and *λ* is a bandwidth parameter proportional to the standard deviation of the signal. It must be pointed out that filtering algorithms inevitably degrade the details, and really plays a vital role in the heart rate recovering. However, it provides a robust HR time series from irregular heartbeats, which were flooded with artifacts. In addition, expanding signals ranging (from 30 bpm to 150 bpm) were discussed in this database, because there is a database for arrhythmia study that has tachycardias or bradycardia, even if it is collected in resting ECGs test. Signals of CNN and RNN were set at a sampling rate of 640 Hz and 128 Hz, respectively, and we input a 0.4 s ECG segment and forecasted following 1.6 s. The result is presented in Table 4.

In the arrhythmia case, M_e_, HR_SD_, HR_MAPE_, and HR_RMSE_ increased significantly compared with the normal heart rhythm database. It is easy to see why the approach performs more poorly. On the one hand, the blurring of details of signals caused by the denoising algorithm lead to the loss of information when removing the artifacts of arrhythmias, such as ST segment, QT interval, and so on, which could be regarded as biometric identification for the actual individuals and has been proven in [41]. On the other hand, even though a large number of the heart beat fragments were used for model training, the individual morphological features of ECGs varied among patients in the database. In the normal heart rhythm database, long-term ECG recordings (over 60,000 s) of every person have been utilized. There are many individual targets tested in the database, but only up to signals of 10 s of each patient can be exploited in this experiment. However, the γ were greater than 0.6, indicating that estimated HR from irregular heartbeat signal has a moderate linear correlation with real HR. Results show that HR recovered from incomplete ECGs with arrhythmias validly.

Unlike on the normal heart rhythm database, results on the arrhythmia database displayed a different highlight. Bi-LSTM were found to be better than TCN in forecasting HR from complex context. In the previous results, Bi-LSTM was inferior to TCN in virtually every sector. However, RNN demonstrated its advantage in arrhythmia cases. With significant differences and small data volumes between patients, whereas TCN performed poorly across all metrics. This may be because TCN needs a larger history for predicting and is limited by the receptive field [30]. In addition, inputting the 0.35 s ECG segment and forecasting following 1.65 s, γ will descend to around 0.3, and M_e_, HR_SD_, HR_MAPE_, and HR_RMSE_ will rise to over 40, indicating that estimated HR has a weak correlation with the ground truth. For a larger range and wider variety of HR, a longer inputting series (0.4 s and above) is needed for prediction.

### 4.4. The Result in More General Cases

In the experiments above, we focused on normal ECGs distributed over a regular range (60–120 bpm) and ECGs from patients with arrhythmias distributed over a wider range (30–150 bpm). They begin with the same position in order to find the best results for predicting HR. In fact, a human’s HR is 20–240 bpm, considering the abnormal situations and the starting position of the heart rhythm should be arbitrary and random. It is worthwhile to explore the performance of iHR estimation in this case. In support of the worst-case scenario, the input signal contains only R and S wave groups. Two uncertain R peak’s positions make it more difficult to predict and confirm the cardiac cycle. Therefore, the model will predict the output in both directions for previous and future moments. In addition, the output of models will include the input directly rather than put the input and output together. We can also take the output in this way in the previous experiments. However, it takes more cost for model training because neurons in the output layer significantly increased, and this is not necessary for the case of unidirectional forecasting. Specifically, we used ECG data from 0.75–1.25 s (0.5 s length) to predict the data from 0–2 s (2s length).

**Figure 7:**
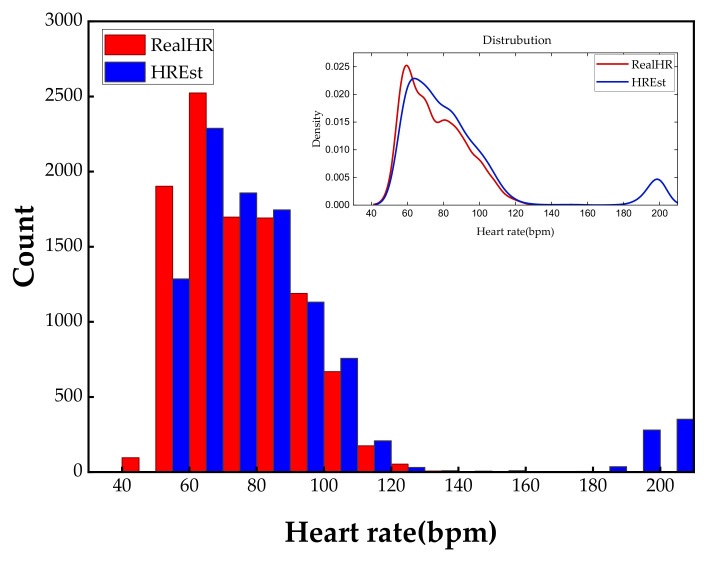
The statistical histograms of real HR and estimated HR in the test set. The red bar represents the ground truth HR, and the blue bar represents the predicted values by TCN. We also plot the kernel density curves of real HR and estimated HR with the red line and blue line, respectively, in the upper right corner.

We show the statistical histogram of the difference between the predicted HR and the real HR in Figure 7 to demonstrate the effectiveness of past and future forecasting approximated by the neural network. It is clear that the HR estimated by the proposed approach has an exactly similar distribution with the ground truth. Furthermore, our approach can predict the HR values more accurately in the range of 60–180 bpm, because majority of the proportion in the dataset are taken up by these frequency parts. Hence, consisting of the perception of machine learning performance, the distribution of the dataset is important in our method. On the contrary, the low-frequency (lower than 60 bpm) and high-frequency (higher than 120 bpm) have unidentical distribution because of the scarcity of these two parts. Compared with the previous experiments where the input sequences start at a constant position, longer duration is needed to be fed into the network to guarantee the performance. Nevertheless, we still achieved a recovery of HR in a crippled cycle. This is the first research to engage in HR prediction and recovery in less than one cycle.

### 4.5. Further Discussion

We experimentally demonstrated the concept of the time series forecasting used in iHR estimation based on 1D ECG signals. PPG, rPPG and other biomedical signals that have the same periodicity as ECG, and could also be predicted and estimated. To some extent, these physiologic index data are more prone to produce a risk of missing integrity for instability in the sensing process. Perhaps it helps build a fast predictive schema to process signals that have a fixed-mode in a very short-time. In addition, 2D images can also be used as input to estimate iHR using the advantage of CNN in extracting image features. However, 2D signal processing creates computation burden and lengthens the computing time significantly. In fact, the model iterated quickly in a desktop computer without GPU acceleration during the 1D data training phase. Once the model converges, getting the predicted result will cost just once forward propagation computation. In other words, the recommended method makes low demands of the computing environment. Thus, the prospects for further deployment of this approach into a sensor or integrated circuit are excellent.

We have discussed the influence of changing the duration of input sequences, and we concluded that predicted results are not credible for inputs no more than 0.3 s. We found that the principal error came from the unusual heart rhythms, as shown in Figure 7. Noticing that there are no existing real HR around 200 bpm, our method produced forecasting results for the corresponding range paradoxically. We observed that the outliers of 2D visualization image formats, such as in Figure 5, and we detected that estimation errors mainly came from the deformation of R peaks. The reason why 0.3 s become a tipping point is that a large number of T waves are misjudged as R waves. Specifically, a 0.3 s duration of the input sequence does not include the T waves, and it is harder to distinguish between R waves with excessive deformation and the T waves with better recovery. If we are able to eliminate the bad effect of other waves on R peaks, perhaps the duration of input sequences can be shorter. Specifically, it is assumed that R waves will not appear for a short period of time after the arrival of the R wave, meaning that a portion of the input sequence will be ignored when calculating HR. This time interval is defined as the skipped window in this paper, and we reproduced the experiment of CNN models using a lag of 0.3 s to predict ECG in the next 0.7 s with a skipped window size of 0.4 s. The reason we chose a window size such as 0.4 s is that the HR is distributed in the range of 60–120 bpm. Compared with previous results, results of new realization outperform previous results in all aspects. γ is improved to 0.89, which indicates that a strong linear correlation between the estimated HR and the real HR. RMSE is reduced to 6.94 from 26.47, and SD_e_ is reduced to 6.76 from 24.0, indicating that HR errors in the predictions are decreased significantly and more concentrated around the real values. In addition, M_e_ is reduced to −1.59 and MAPE is reduced to 6.12%, which demonstrates that the accuracy of forecasting results has been improved. In general, introducing an appropriate skipped window cuts down the minimum duration required for the input sequence.

We have further shortened the duration of input sequences and found that γ was greater than 0.7 and RMSE and SD_e_ were less than 10 when input duration is reduced to 0.2 s or less. Although the amplitude of the predicted R wave is already very low in this case, the values of the metrics indicate that the proposed approach is still effective. Nevertheless, extremely short-time estimation has to be implemented under the premise that the HR diversity of the dataset is limited to a small range and starts at a constant position. Therefore, the proposed method is more applicable for estimating the resting HR (usually 60–100 bpm) or some personalized predictions.

Analysis of the heart rate variability (HRV) time series is particularly crucial in clinical diagnosis and treatment of patients. Wessel et al., proposed a short-term forecasting approach for quantifying global short-term predictability based on symbolic dynamics and finite-time growth rates in [42,43]. It presented relatively good results in HRV analysis. However, the HRV analysis relies on tachograms, which are obtained by calculating beat-to-beat intervals. Therefore, the inputs of these methods should last for more than one complete cycle. While our research focus on ECGs on a smaller timescale, i.e., ECG signal less than one cardiac cycle, which makes it difficult to estimate HR as accurate as the aforementioned HRV method.

CNN and RNN both showed real strength of predictive ability. CNN outperforms in a long-term database with less computation efforts, while RNN offers stronger adaptability in diverse and small data cases. Correspondingly, CNN is suited to personal prediction and RNN is applicable for more accurate detection of new samples. Therefore, mounting researchers offer perfect fusion of CNN and RNN [44,45,46]. Taking advantage of complementarities among models, the approach is regarded to achieve deep learning with defective data in the future, such as a small number of data or unsupervised data [47,48].

## 5. Conclusions

In this paper, we proposed an approach for iHR estimation based on CNN and RNN from 1D ECG data. Conventional methods for HR estimation rely on stable data lasting for several cycles, while we focused on estimating HR in a very short time or even less than one cardiac cycle. The method takes a time series model to predict the output sequence in the near future and estimates the corresponding HR through QRS detection. The DL models were trained with fragmentary input sequences derived from long-time ECG signals. Our experiments on the MIT-BIH Normal Sinus Rhythm Database and A Large Scale 12-lead Electrocardiogram Database for Arrhythmia Study validate that the method is technically feasible. The result of the Pearson correlation coefficient is greater than 0.7, which shows a stronger linear correlation between real HR and estimated HR. We also analyzed some basic factors that may affect the performance of our method. It is observed that unilateral sequence length changes make little difference in the prediction results, while the input duration plays a key role in iHR estimation. As input duration decreases, the performance degradation emerged for both CNN and RNN. Under the condition that the starting position of the input sequence is determined, valid results can be predicted with the shortest input of about 0.35 s, and the timing length will be able to reduce to 0.2 s considering a specific situation when a human is at rest. In a more general case, output data that were predicted bidirectionally and the proposed approach still works well.

The approach could be improved in the future. We can estimate HR from ECG in advance, but the measurement result is influenced by the measurement environment of a human, which means that accurate results came more from normal heart rhythms. It is still more applicable to estimate HR in resting state or some personalized predictions and does not apply to HRV time series analysis recently. Yet the approach would be valuable in many areas. For instance, heart rate, respiration, and temperature are highly correlated, and short-time heart rate estimation will benefit the measurement of additional indicators. Furthermore, a data-driven biometrics framework depends on the quality of signals, the approach can provide replacements for poor-quality signals, and makes it even more useful. The approach makes low demands of the computing environment, data integrity, and signal quality. In addition, when combined with CNN and RNN, it is achievable with the use of transfer learning with fine-tuning [49]. Thus, the prospects for further deployment of this approach into a mobile sensor are excellent. 

## Figures and Tables

**Table 1:** iHR estimation performance comparisons on the MIT-BIH Normal Sinus Rhythm Database at various sampling frequencies (64–1024 Hz).

DL Models	Sampling Rate (f_s_)	M_e_ (bpm)	HR_SD_ (bpm)	HR_MAPE_	HR_RMSE_ (bpm)	γ
Bi-LSTM (RNN)	0.5	1.98	6.19	5.82%	6.51	0.91
	1	−0.25	5.91	5.22%	5.91	0.92
	2	0.70	6.03	5.39%	6.07	0.92
	3	−0.14	5.97	5.28%	5.97	0.92
	4	0.32	6.29	5.47%	6.30	0.91
	5	−0.34	6.04	5.26%	6.05	0.91
	6	0.43	6.24	4.98%	6.25	0.91
	7	0.71	6.26	5.58%	6.30	0.91
	8	0.50	6.03	5.31%	6.05	0.91
TCN (CNN)	0.5	1.01	6.18	5.43%	6.27	0.91
	1	0.03	5.90	5.18%	5.90	0.92
	2	−0.37	5.74	5.10%	5.75	0.93
	3	−0.58	5.67	5.07%	5.70	0.93
	4	−0.73	5.79	5.17%	5.83	0.92
	5	−0.53	5.62	4.96%	5.64	0.93
	6	−0.61	5.78	5.17%	5.81	0.92
	7	−0.98	5.66	5.06%	5.75	0.93
	8	−0.76	5.79	5.17%	5.84	0.92

**Table 2:** Some key parameters of RNN models with the proportion of the input sequence *α* = 0.5.

Layers	No. of Neurons	Dropout Rate
LSTM1	60	0.6
LSTM2	30	0.4
LSTM3	20	0.3

**Table 3:** Some key parameters of CNN models with the proportion of the input sequence *α* = 0.5.

Modules	Filter Length/Dropout Rate	Dilation Factors
ResBlock1	128/0.2	1
ResBlock2	128/0.2	2
ResBlock3	64/0.2	4
ResBlock4	64/0.2	8
ResBlock5	32/0.2	16
ResBlock6	32/0.2	32
ResBlock7	16/0.2	64
ResBlock8	16/0.2	128
ResBlock9	8/0.2	256
ResBlock10	8/0.2	320

**Table 4:** Inputting a 0.4 s ECG segment and forecasting following 1.6 s, the iHR estimation performance comparisons on the A Large Scale 12-lead Electrocardiogram Database at different models (CNN and RNN) are shown. All the ECGs were filtered by the NLM method, and signals of CNN and RNN were set at a sampling rate of 640 Hz and 128 Hz.

DL Models	M_e_ (bpm)	HR_SD_ (bpm)	HR_MAPE_	HR_RMSE_ (bpm)	γ
Bi-LSTM	−2.66	25.24	21.77%	25.38	0.68
TCN	−4.01	26.22	23.37%	26.52	0.65

## Data Availability

The data that support the fundings of this study are openly available in PhysioNet. MIT-BIH Normal Sinus Rhythm database on https://doi.org/10.13026/C2NK5R, reference number [37], and A Large Scale 12-lead Electrocardiogram Database for Arrhythmia Study database on https://doi.org/10.13026/wgex-er52, accessed on 24 August 2022, reference number [38,39].
